# Clinical use of exhaled volatile organic compounds in pulmonary diseases: a systematic review

**DOI:** 10.1186/1465-9921-13-117

**Published:** 2012-12-21

**Authors:** Kim DG van de Kant, Linda JTM van der Sande, Quirijn Jöbsis, Onno CP van Schayck, Edward Dompeling

**Affiliations:** 1 Department of Pediatric Pulmonology, School for Public Health and Primary Care (CAPHRI), Maastricht University Medical Center (MUMC), P.O. Box 5800, 6202, AZ, Maastricht, the Netherlands; 2 Department of General Practice, CAPHRI, MUMC, P.O. Box 5800, 6202, AZ, Maastricht, the Netherlands

**Keywords:** VOCs, Asthma, COPD, Lung cancer, Cystic fibrosis, Airway inflammation, Biomarkers

## Abstract

There is an increasing interest in the potential of exhaled biomarkers, such as volatile organic compounds (VOCs), to improve accurate diagnoses and management decisions in pulmonary diseases. The objective of this manuscript is to systematically review the current knowledge on exhaled VOCs with respect to their potential clinical use in asthma, lung cancer, chronic obstructive pulmonary disease (COPD), cystic fibrosis (CF), and respiratory tract infections. A systematic literature search was performed in PubMed, EMBASE, Cochrane database, and reference lists of retrieved studies. Controlled, clinical, English-language studies exploring the diagnostic and monitoring value of VOCs in asthma, COPD, CF, lung cancer and respiratory tract infections were included. Data on study design, setting, participant characteristics, VOCs techniques, and outcome measures were extracted. Seventy-three studies were included, counting in total 3,952 patients and 2,973 healthy controls. The collection and analysis of exhaled VOCs is non-invasive and could be easily applied in the broad range of patients, including subjects with severe disease and children. Various research groups demonstrated that VOCs profiles could accurately distinguish patients with a pulmonary disease from healthy controls. Pulmonary diseases seem to be characterized by a disease specific breath-print, as distinct profiles were found in patients with dissimilar diseases. The heterogeneity of studies challenged the inter-laboratory comparability. In conclusion, profiles of VOCs are potentially able to accurately diagnose various pulmonary diseases. Despite these promising findings, multiple challenges such as further standardization and validation of the diverse techniques need to be mastered before VOCs can be applied into clinical practice.

## Review

### Introduction

#### Background and aim

Pulmonary diseases are important causes of morbidity in both adults and children [1,2]. The diverse pulmonary diseases go along with clinical challenges. In adults, lung cancer is one of the leading causes of death worldwide. It is often diagnosed at an advanced stage when successful treatment is difficult [3]. Furthermore, chronic obstructive pulmonary disease (COPD) and asthma are prevalent lung diseases that account for a major burden on society in terms of morbidity and health care costs. Early diagnosis and close monitoring of both diseases are important for proper treatment decisions, optimal disease control and prognosis. However, the available clinical tools are not always fulfilling. In young children, a reliable asthma diagnosis is difficult as there are no tools available to discriminate between true asthmatics and children with transient, virus-induced symptoms. On account of these clinical challenges, there is a continuous search for techniques that can improve accurate diagnoses and management decisions. A potential non-invasive technique is the analysis of volatile biomarkers in exhaled breath, so called volatile organic compounds (VOCs). In this manuscript we systematically review the current knowledge on VOCs regarding their potential clinical use in pulmonary diseases.

#### The origin of exhaled volatile organic compounds

**Figure 1:**
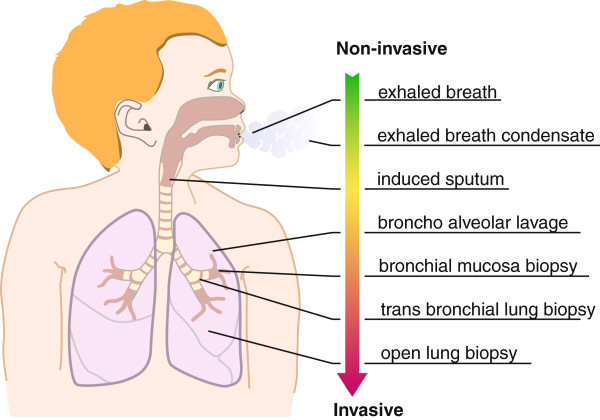
**Techniques to assess airway inflammation and oxidative stress.** There are various methods to measure airway inflammation and oxidative stress ranging from completely non-invasive (exhaled breath analysis) to very invasive (open lung biopsy).

Asthma, COPD, Cystic Fibrosis (CF), and lung cancer are characterized by inflammation and oxidative stress. Monitoring of airway inflammation and oxidative stress can be helpful in the diagnosis and monitoring of these diseases. Current available techniques to directly measure inflammation and oxidative stress in the airways are bronchoscopy, bronchoalveolar lavage and biopsy. These techniques are too invasive for repeated routine use, especially in children. The need for non-invasive analysis of inflammation and oxidative stress in the lungs has led to increasing interest in exhaled breath analysis (Figure 1). Fractional exhaled Nitric Oxide (FeNO) is the most extensively studied marker in exhaled breath. Although the analysis of FeNO might be a helpful clinical tool in some pulmonary diseases, it has several limitations. For example in asthma, FeNO is especially a marker of allergic inflammation and therefore of limited use in non-allergic patients [4]. Consequently, additional exhaled biomarkers were studied. Next to non-volatile biomarkers that can be assessed in exhaled breath condensate, the analysis of exhaled VOCs gained popularity. VOCs are a diverse group of carbon-based chemicals that are volatile at room temperature. The source of exhaled VOCs can be exogenous or endogenous. Some VOCs can be taken up as pollutants from the environment via the skin or by inhalation or ingestion. Subsequently, these compounds are metabolized and exhaled. Other VOCs are formed in the body during several (patho)physiological processes [5,6]. An important group of endogenously formed VOCs are hydrocarbons that are formed by lipid peroxidation. During the inflammatory process, Reactive Oxygen Species (ROS) are produced by inflammatory cells. Subsequently, ROS react with lipid membrane structures and cause degradation of polyunsaturated fatty acids. As a result several stable breakdown products including hydrocarbons are formed [5,6]. Besides hydrocarbons, other VOCs can be identified, including nitrogen, oxygen or sulphur containing compounds. These VOCs can be formed by bacteria or during (patho)physiological processes in the liver, kidneys, and pancreas [5,6]. As soon as VOCs are formed, they are either further oxidized into smaller components due to enhanced activity of enzymes (such as cytochrome P450 oxidase), or they directly enter the bloodstream [7]. Subsequently, VOCs are excreted into breath. Early findings of distinct VOCs in diseased people (e.g. with diabetes or cirrhosis) compared to healthy controls stimulated investigators to elucidate the clinical potential of exhaled VOCs in pulmonary diseases [5]. Since exhaled VOCs are formed during inflammatory processes, the analysis of VOCs may be a promising non-invasive technique to directly monitor inflammation and oxidative stress in the airways. This information might be of help in the diagnosis and monitoring of pulmonary diseases.

#### Technical analysis of exhaled breath

**Figure 2:**
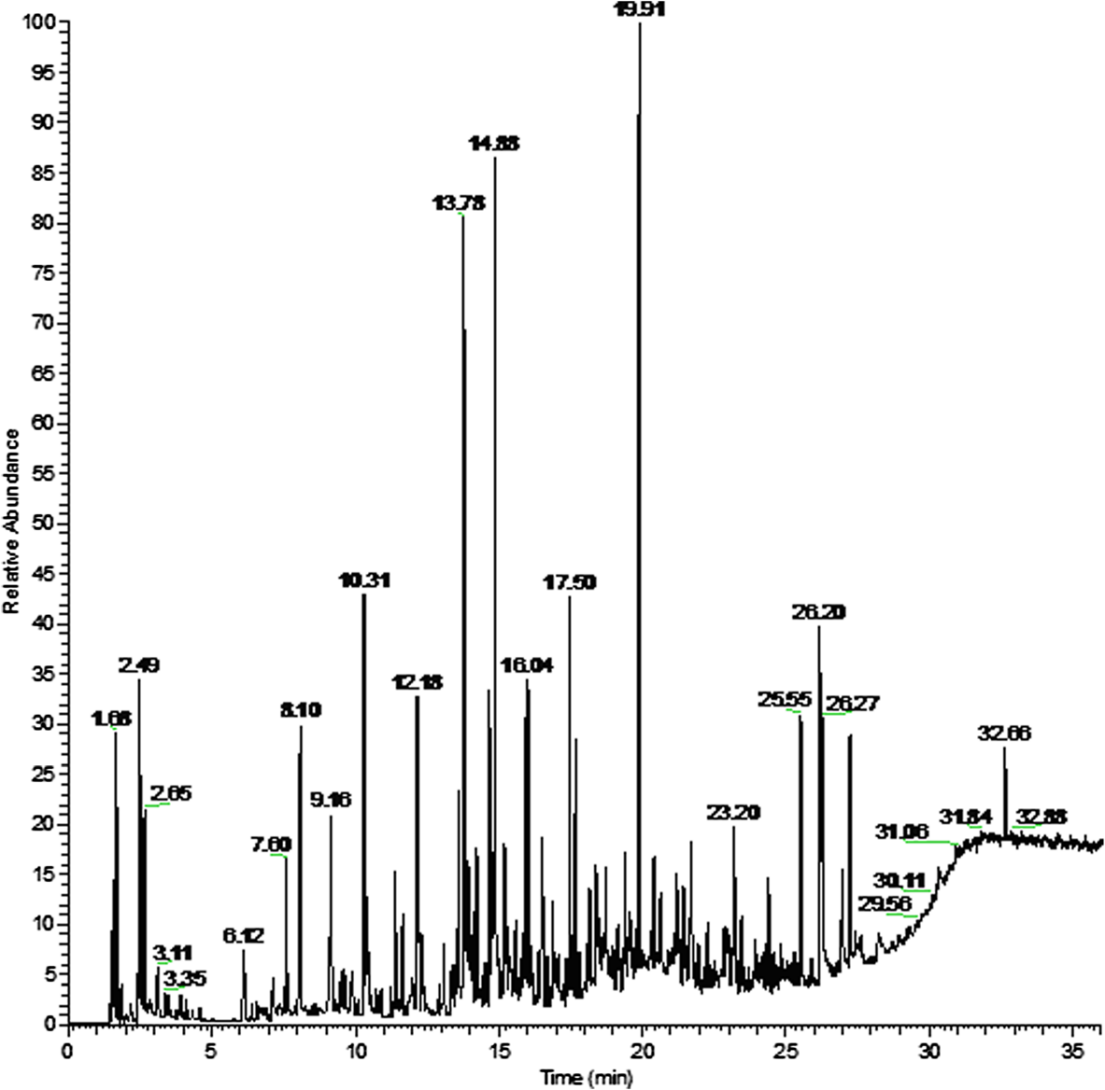
**Breath-print of VOCs by gas chromatography.** With the gas chromatography (GC) technique, exhaled breath is collected and temporarily stored in e.g. gas-tight syringes, glass bulbs, inert bags, or metal containers. Once the VOCs are collected and temporarily trapped, they can be released for analysis. This is often performed by solvent or thermal desorption. Subsequently, the analysis of individual molecular components can be assessed by GC usually followed by mass spectrometry (GC-MS) or flame ionization detection (GC-FID). The diverse VOCs are separated and quantified by using their specific compound characteristics. Distinct VOCs have dissimilar progression rates and reach the end of the GC column at different time points; the retention time. Based on their retention time, VOCs can be identified in a mass-spectra library. The figure demonstrates an example of a chromatogram of a breath sample analyzed with GC. The retention time (in minutes) is stated on the x axis, while the y axis shows the relative abundance of various compound signals. *Published in Robroeks* et al. *Pediatr Res 2010 *[14].

**Figure 3:**
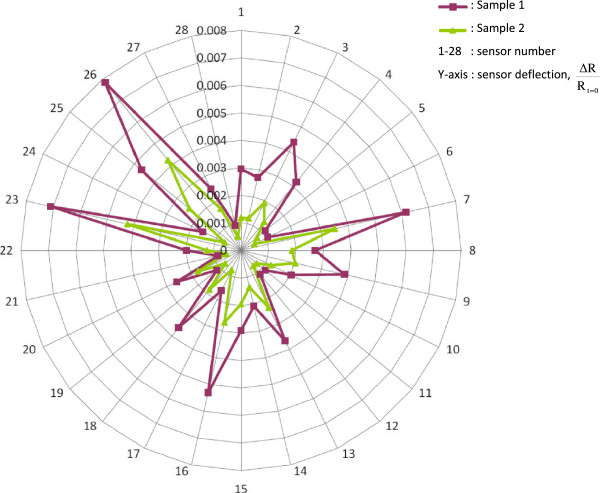
**Breath-print of VOCs by the electronic Nose.** Breath samples can also be analyzed using an eNose. The eNose consists of an array of nanosensors. When these sensors are exposed to a mixture of VOCs, a change in their electrical resistance is induced, leading to the production of a ‘breath-print’. This breath-print represents the complex mixture of exhaled VOCs and can be used for pattern-recognition algorithms in multiple diseases. A limitation of the eNose is that it is unable to analyze individual VOCs. In the figure two exhaled breath-prints analyzed with the eNose are demonstrated (purple line represents sample 1, green line represents sample 2). The y axis represents the change in resistance (Δ R/Rt = 0) of each of the 28 sensors (1–28). *Courtesy: Paul Brinkman, Niki Fens, Peter Sterk, University of Amsterdam, the Netherlands.*

There are multiple techniques described to collect, detect, and analyze exhaled VOCs [6,8,9]. The most commonly used techniques are gas chromatography (GC), which is the gold standard, and the electronic nose (eNose). With the GC-technique, exhaled breath is firstly collected and temporarily stored (e.g. in inert bags or sorption tubes). After a desorption phase, individual VOCs can be assessed by GC usually followed by mass spectrometry (GC-MS) or flame ionization detection (GC-FID) [6]. The diverse VOCs are first separated based on their chemical properties and consecutively ionized and separated by their mass-to-charge (m/z) ratio (Figure 2). Breath samples can also be analyzed using an eNose [9]. The eNose consists of an array of nanosensors. When these sensors are exposed to a mixture of VOCs, a change in their electrical resistance is induced, leading to the production of a ‘breath-print’ (Figure 3). This breath-print represents the complex mixture of exhaled VOCs and can be used for pattern-recognition algorithms in multiple diseases [10-13]. A limitation of the eNose is that it is unable to analyze individual VOCs. In addition to GC and the eNose, other techniques that are used to study VOCs in pulmonary diseases include; proton transfer reaction mass spectrometry (PTR-MS), selected ion flow tube mass spectrometry (SIFT-MS), ion mobility spectrometry (IMS), laser spectroscopy, colorimetric sensor array, and gold nano particles sensors (GNPs).

## Materials and methods

### Data sources and search criteria

A systematic literature search was performed until July 2012 in PubMed, EMBASE, and the Cochrane Central Register of Controlled trials. Keywords/Mesh terms included: asthma, chronic obstructive pulmonary disease, COPD, cystic fibrosis, lung cancer, pulmonary disease, respiratory infection, combined with: volatile organic compounds, VOC, VOCs, exhaled breath or electronic nose. Reference lists were reviewed for additional references.

### Study selection and data extraction

**Figure 4:**
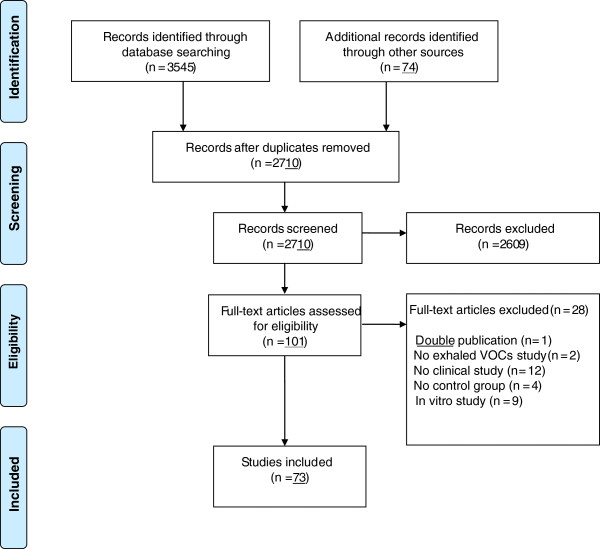
**Flow-chart of literature search.** Summary of evidence search and selection according to the Prisma flow-chart [16]. Abbreviations: VOCs = Volatile Organic Compounds.

Figure 4 illustrates a flow-chart of the study selection [16]. Controlled, clinical studies, with full text in English, exploring the diagnostic and monitoring value of VOCs in asthma, COPD, CF, lung cancer and respiratory tract infections were included. In vitro studies were excluded. Data on study design, setting, participant characteristics, VOCs techniques, and outcome measures were extracted. Due to expected heterogeneity of studies, no single scale was used for excluding studies on basis of quality. Instead, per study, criteria that are of importance to examine the validity are described in Table 1.

**Table 1:** Characteristics of included studies on VOCs in pulmonary diseases

**Author (year)**	**Design**	**Comparison**	**Sample size**	**Setting**	**Technique**	**Outcome measure**	**Ref.**
Caldeira (2011)	Cross-sectional	Asthma vs. controls	35 children with asthma, 15 healthy controls	Hospital D. Pedro, Aveiro (Portugal)	GC-MS	Set of 44 VOCs	[17]
Caldeira (2012)	Cross-sectional	Asthma vs. controls	32 children with allergic asthma, 27 healthy controls	Hospital D. Pedro, Aveiro (Portugal)	GC-MS	VOCs profile	[18]
Dallinga (2010)	Cross-sectional	Asthma vs. controls	63 children with asthma, 57 healthy controls	Maastricht University MC	GC-MS	VOCs profile	[19]
				(the Netherlands)			
Dragonieri (2007)	Cross-sectional	Asthma vs. controls	10 patients with mild asthma, 10 patients with severe asthma, 20 healthy controls	Leiden University MC (the Netherlands)	eNose	VOCs profile	[10]
Ibrahim (2011)	Cross-sectional	Asthma vs. controls	35 patients with asthma, 23 healthy controls	Wythenshawe Hospital, Manchester (UK)	GC-MS	VOCs profile	[20]
Lärstad (2007)	Cross-sectional	Asthma vs. controls	13 patients with asthma, 14 healthy controls	Göteborg University (Sweden)	GC-FID	Ethane, Pentane, Isoprene	[21]
Montuschi (2010)	Cross-sectional	Asthma vs. controls	27 patients with asthma, 24 healthy controls	Istituto Dermopatico dell’ Immacolata, Rome (Italy)	eNose, GC-MS	VOCs profile	[22]
Olopade (1997)	Cross-sectional Short follow-up in acute asthma	Asthma vs. controls	12 patients with acute asthma, 11 patients with stable asthma, 17 healthy controls	University of Ilinois Hospital, Chicago (USA)	GC-FID	Pentane	[23]
Paredi (2000)	Cross-sectional	Asthma vs. controls	26 patients with asthma, 14 healthy controls	National Heart and Lung Institute, Imperial College, London (UK)	GC-FID	Ethane	[24]
Basanta (2010)	Cross-sectional	COPD vs. smokers	20 patients with COPD, 6 healthy smokers	Wytenshawe Hospital, Manchester (UK)	GC-DMS	VOCs profile	[25]
Cristescu (2011)	Cross-sectional	Emphysema vs. No emphysema	204 (former) smokers (43 with emphysema/COPD)	Radboud University, Nijmegen (the Netherlands)	PTR-MS	Mass-spectra	[26]
Fens (2009)	Cross-sectional	COPD vs. asthma vs. controls	30 patients with COPD, 20 patients with asthma, 20 non-smoking controls, 20 smoking controls	Academic MC Amsterdam; Haga Teaching Hospital, The Hague; Albert Schweitzer Hospital, Dordrecht (the Netherlands)	eNose	VOCs profile	[15]
Fens (2011)	Cross-sectional	COPD vs. asthma	40 patients with COPD, 21 patients with fixed asthma, 39 patients with classic asthma	Academic MC Amsterdam; Haga Teaching Hospital, The Hague; Albert Schweitzer Hospital, Dordrecht (the Netherlands)	eNose	VOCs profile	[27]
Hattesohl (2011)	Cross-sectional Follow up after treatment	COPD vs. controls	10 patients with COPD with AAT deficiency, 23 patients with COPD without AAT deficiency, 10 healthy controls	Phillipps University, Marburg (Germany)	eNose	VOCs profile	[28]
Hauschild (2012)	Cross-sectional	COPD vs. controls	30 patients with COPD, 54 patients with COPD + BC, 35 healthy controls	Max Planck Institute for Informatics, Saarbrücken (Germany)	IMS	VOCs profile	[29]
Paredi (2000)	Cross-sectional	COPD vs. controls	22 patients with COPD, 14 healthy controls	National Heart and Lung Institute, Imperial College, London (UK)	GC-FID	Ethane	[30]
Phillips (2012)	Cross-sectional	COPD vs. controls	119 patients with COPD, 63 healthy controls	Swansea University, Swansea (UK)	GC-MS	VOCs profile	[31]
Timms (2012)	Cross-sectional	COPD vs. asthma vs. controls	17 patients with COPD, 20 patients with asthma, 7 healthy controls	University of New South Wales, Sydney (Australia)	eNose	VOCs profile	[32]
Van Berkel (2010)	Cross-sectional	COPD vs. controls	66 patients with COPD, 15 steroid naïve COPD patients, 45 healthy controls	Maastricht University MC (the Netherlands)	GC-MS	VOCs profile	[33]
Barker (2006)	Cross-sectional	CF vs. controls	20 patients with CF, 20 healthy controls	Aachen CF center (Germany)	GC-MS	Set of 12 VOCs	[34]
Enderby (2009)	Cross-sectional	CF vs. asthma	16 patients with CF, 21 patients with asthma	University Hospital of North Staffordshire,Stoke-on-Trent (UK)	SIFT-MS	Hydrogen cyanide	[35]
Gilchrist (2012)	Cross-sectional	CF with- vs. CF without *Ps.* infection	8 CF patients with *Ps.* infection, 7 CF patients without *Ps.* infection	University Hospital of North Staffordshire,Stoke-on-Trent (UK)	SIFT-MS	Hydrogen cyanide	[36]
Kamboures (2005)	Cross-sectional	CF vs. controls	20 patients with CF, 23 healthy controls	University of California, Irvine (USA)	GC-MS	Carbonyl sulphide, Dimethyl sulphide, Carbon disulphide	[37]
McGrath (2000)	Cross-sectional Follow up after treatment	CF during- vs. CF after exacerbation vs. controls	12 patients with CF, 12 healthy controls	Queen’s University, Belfast (UK)	GC-MS	Isoprene	[38]
Paredi (2000)	Cross-sectional	CF vs. controls	23 patients with CF, 14 healthy controls	National Heart and Lung Institute, Imperial College, London (UK)	GC-FID	Ethane	[39]
Robroeks (2010)	Cross-sectional	CF vs. controls	48 patients with CF, 57 healthy controls	Maastricht University MC (the Netherlands)	GC-MS	VOCs profile	[14]
Shestivska (2011)	Cross-sectional	CF vs. controls	28 patients with CF, 9 healthy controls	Academy of Science of the Czech Republic, Prague (Czech Republic)	GC-MS	Methyl thiocyanate	[40]
Bajtarevic (2009)	Cross-sectional	LC vs. controls	285 patients with LC, 472 healthy controls	Innsbruck Medical University (Austria)	PTR-MS, GC-MS	VOCs profile	[41]
Buszewski (2012)	Cross-sectional	LC vs. controls	29 patients with LC, 44 healthy controls	Nicolaus Copernicus University, Torun (Poland)	GC-MS	Set of multiple VOCs	[42]
Crohns (2009)	Cross-sectional Follow up after treatment	LC vs. controls	11 patients with LC, 30 healthy controls	Tampere University Hospital (Finland)	GC-MS	Pentane	[43]
D’Amico (2010)	Cross-sectional	LC vs. no LC vs. controls	28 patients with LC, 28 patients with diverse lung diseases (e.g. COPD (n = 16), bronchitis), 36 healthy controls	C. Forlanini Hospital, Rome (Italy)	eNose (GC-MS)	VOCs profile	[44]
Di Natale (2003)	Cross-sectional	LC vs. controls	35 patients with LC, 9 post-surgical LC patients, 18 healthy controls	C. Forlanini Hospital, Rome (Italy)	eNose (GC-MS)	VOCs profile	[11]
Dragonieri (2009)	Cross-sectional	LC vs. COPD vs. controls	10 patients with NSCLC, 10 patients with COPD, 10 healthy controls	Leiden University MC (the Netherlands)	eNose	VOCs profile	[12]
Fuchs (2010)	Cross-sectional	LC vs. controls	12 patients with LC, 12 healthy smokers, 12 healthy controls	University Rostock (Germany)	GC-MS	Set of 10 volatile aliphatic aldehydes	[45]
Gaspar (2009)	Cross-sectional	LC vs. controls	18 patients with LC, 10 healthy controls	University of Lisbon (Portugal)	GC-MS	VOCS profile	[46]
Gordon (1985)	Cross-sectional	LC vs. controls	12 patients with LC, 9 healthy controls	Michael Reese Hospital, Chicago (USA)	GC-MS	Set of 22 VOCs	[47]
Kischkel (2010)	Cross-sectional	LC vs. controls	31 patients with LC, 31 healthy smokers, 31 healthy controls	University of Rostock (Germany)	GC-MS	Set of 42 VOCs	[48]
Ligor (2009)	Cross-sectional	LC vs. controls	65 patients with LC, 31 healthy controls	Innsbruck Medical University (Austria)	GC-MS	Set of 103 VOCs	[49]
Machado (2005)	Cross-sectional	LC vs. no LC vs. controls	28 patients with LC, 57 patients with diverse lung diseases (e.g. COPD (n = 12), asthma (n = 11), CBD), 50 healthy controls	Cleveland Clinic (USA)	eNose (GC-MS)	VOCs profile	[13]
Mazzone (2007)	Cross-sectional	LC vs. no LC vs. controls	49 patients with NSCLC, 73 patients with diverse lung diseases (e.g. COPD (n = 18), sarcoidosis), 21 healthy controls	Cleveland Clinic (USA)	Colorimetric sensor array	VOCs profile	[50]
Mazzone (2012)	Cross-sectional	LC vs. controls	92 patients with LC, 59 healthy smokers, 78 patients with diverse lung diseases (e.g. COPD (n = 8))	Cleveland Clinic (USA)	Colorimetric sensor array	VOCs profile	[51]
Peng (2009)	Cross-sectional	LC vs. controls	40 patients with LC, 56 healthy controls	Rambam Health Care Campus, Haifa (Israel)	GNPs GC-MS	VOCs profile	[52]
Peng (2010)	Cross-sectional	LC vs. controls	30 patients with PLC, 22 healthy controls	Rambam Health Care Campus, Haifa (Israel)	GNPs GC-MS	VOCs profile	[53]
Phillips (1999)	Cross-sectional	LC vs. no LC	108 patients with abnormal chest radiograph (60 patients with LC)	Penn State MC, Hershey (USA); Hammersmith Hospital, London (UK); St. Vincent’s MC, New York (USA)	GC-MS	VOCs profile	[54]
Phillips (2003)	Cross-sectional	LC vs. no LC vs. controls	178 patients with abnormal chest radiograph (87 patients with LC), 41 healthy controls	Charing Cross Hospital, London (UK); Columbia Presbyterian MC/New York University MC/St. Vincent’s MC, New York (USA); Penn State MC, Hershey (USA)	GC-MS	VOCs profile	[7]
Phillips (2007–2008)	Cross-sectional	LC vs. controls	193 patients with PLC, 211 (former) healthy smokers	Harper Hospital, Detroit; New York University MC/Columbia University MC/Weill Medical College of Cornell University, New York (USA); University of California, Los Angeles; Danbury Hospital, Connecticut (USA).	GC-MS	VOCs profile	[55], [56]
Poli (2005)	Cross-sectional Short follow-up in LC	LC vs. COPD vs. controls	36 patients with NSCLC, 25 patients with COPD, 35 healthy smokers, 50 healthy non-smokers	University of Parma (Italy)	GC-MS	Set of 13 VOCs	[57]
Poli (2008)	Follow-up	LC before vs. after surgery	36 patients with NSCLC, 50 healthy controls	University of Parma (Italy)	GC-MS	Set of 12 VOCs	[58]
Poli (2010)	Cross-sectional	LC vs. controls	40 patients with NSCLC, 38 healthy controls	University of Parma (Italy)	GC-MS	Set of 7 aldehydes	[59]
Preti (1988)	Cross-sectional	LC vs. controls	10 patients with LC, 16 healthy controls	University Hospital Pennsylvania, Philadelphia (USA)	GC-MS	Aniline, o-Toluidine	[60]
Rudnicka (2011)	Cross-sectional	LC vs. controls	23 patients with LC, 30 healthy controls	Nicolaus Copernicus University, Torun (Poland)	GC-MS	Set of 55 VOCs	[61]
Skeldon (2006)	Cross-sectional	LC vs. no LC vs. controls	12 patients with LC, 40 patients with diverse lung diseases, 58 healthy controls	Ninewells Hospital, Dundee (UK)	Laser absorption spectroscopy	Ethane	[62]
Song (2010)	Cross-sectional	LC vs. controls	43 patients with NSCLC, 41 healthy controls	Anhui Medical University, Hefei, Anhui (China)	GC-MS	1-butanol, 3-hydroxy-2-butanone	[63]
Steeghs (2007)	Cross-sectional	LC vs. controls	11 patients with LC, 57 healthy smokers	Radboud University, Nijmegen (the Netherlands)	PTR-MS	Mass-spectra	[64]
Ulanowska (2011)	Cross-sectional	LC vs. controls	137 patients with LC, 143 healthy controls	Nicolaus Copernicus University, Torun (Poland)	GC-MS	VOCs profile	[65]
Wehinger (2007)	Cross-sectional	LC vs. controls	17 patients with PLC, 170 healthy controls	Innsbruck Medical University (Austria)	PTR-MS	Mass-spectra	[66]
Westhoff (2009)	Cross-sectional	LC vs. controls	32 patients with LC, 54 healthy controls	Hemer Lung Hospital (Germany)	IMS	VOCs profile	[67]
Chapman (2012)	Cross-sectional	MPM vs. ARD vs. controls	20 patients with MPM, 18 patients with ARD, 42 healthy controls	St Vincent and Prince of Wales Hospital, Sydney (Australia)	eNose	VOCs profile	[68]
Gennaro (2010) Dragonieri (2012)	Cross-sectional	MPM vs. no MPM	13 patients with MPM, 13 subjects with long-term asbestos exposure, 13 healthy controls	University of Bari Aldo Moro, Bari (Italy)	eNose, GC-MS	VOCs profile	[69], [70]
Chambers (2009)	Cross-sectional	A. fumigatus vs. controls	32 patients with diverse lung diseases (e.g. asthma (n = 11), CF (n = 6), COPD (n = 3), 10 neutropenic patients, 14 healthy controls	University of Christchurch (New Zealand)	GC-MS	2-Pentylfuran	[71]
Hanson (2005)	Cross-sectional	VAP vs. no VAP	19 patients with + VAP score, 19 patients with - VAP score	University of Pennsylvania, Philadelphia (USA)	eNose	VOCs profile	[72]
Hockstein (2004)	Cross-sectional	VAP vs. no VAP	13 ventilated patients with VAP, 12 ventilated patients without VAP	University of Pennsylvania, Philadelphia (USA)	eNose	VOCs profile	[73]
Hockstein (2005)	Cross-sectional	VAP vs. no VAP	15 patients with + VAP score, 29 patients with - VAP score	University of Pennsylvania, Philadelphia (USA)	eNose	VOCs profile	[74]
Kanoh (2005)	Cross-sectional Short follow-up in ILD patients	ILD vs. controls	34 patients with ILD, 16 healthy controls	National Defense Medical College, Saitama (Japan)	GC-FID	Ethane	[75]
Kolk (2012)	Cross-sectional	TB vs. no TB	171 patients suspected of TB	Royal Tropical Institute, Amsterdam (the Netherlands); Desmond Tutu TB Centre, Cape Town (South Africa)	GC-MS	VOCs profile	[76]
Phillips (2007)	Cross-sectional	TB vs. no TB vs. controls	42 patients suspected of TB, 59 healthy controls	Bellevue Hospital, New York (USA)	GC-MS	VOCs profile	[77]
Phillips (2010)	Cross-sectional	TB vs. no TB	226 patients suspected of TB	University of California, San Diego (USA); University of Santo Tomas, Manila (Philippines), De La Salle University Hospital, Cavite (Philippines), East London Tuberculosis Service (UK)	GC-MS	VOCs profile	[78]
Phillips (2012)	Cross-sectional	TB vs. controls	130 patients with TB, 121 healthy controls	University of Santo Tomas, Manila (Philippines); De La Salle University Hospital, Cavite (Philippines); Homerton University Hospital, London (UK); Hinduja Hospital, Mumbai (India)	GC-SAW	VOCs profile	[79]
Syhre (2009)	Cross-sectional	TB vs. controls	10 patients with TB, 10 healthy controls	Otago University, Christchurch (New Zealand); Modilon Hospital, Madang (Papua New Guinea)	GC-MS	Methyl nicotinate	[80]
Scholpp (2002)	Cross-sectional	Critically ill patients vs. controls	65 critically ill patients (n = 19 with head injury, n = 13 with ARDS, n = 33 at risk of ARDS), 10 healthy controls	University Hospital of Freiburg (Germany)	GC-FID, GC-MS	Acetone Isoprene, n-Pentane	[81]
Schubert (1998)	Cross-sectional Short follow-up in VAP patients	ARDS vs. no ARDS	19 critically ill patients with ARDS, 18 critically ill patients without ARDS	University Hospital of Freiburg (Germany)	GC-FID, GC-MS	Acetone Isoprene, n-Pentane	[82]

**Abbreviations:** AAT deficiency = Alpha 1-antitrypsin deficiency; A. fumigatus = Aspergillus fumigatus; ARD = Benign Asbestos-Related Diseases; ARDS = Acute Respiratory Distress Syndrome; BC = Bronchial Carcinoma; CBD = Chronic pulmonary Beryllium Disease; CF = Cystic Fibrosis; Classic asthma = Asthmatics with reversible airway obstruction; COPD = Chronic Obstructive Pulmonary Disease; DMS = Differential Mobility Spectrometry; eNose = electronic Nose; FID = Flame Ionization Detector; Fixed asthma = Asthmatics with fixed airway obstruction; GC = Gas Chromatography; GNPs = Gold Nano Particles sensors; ILD = Interstitial Lung Disease (e.g. sarcoidosis, idiopathic pulmonary fibrosis, cryptogenic organizing pneumonia); IMS = Ion Mobility Spectrometry; LC = Lung Cancer; MC = Medical Centre; MPM = Malignant Pleural Mesothelioma; MS = Mass Spectrometry; NSCLC = Non-Small Cell Lung Cancer; OFD = On-Fiber-Derivatization; *P*. infection = *Pseudomonas aeruginosa* infection; PLC = Primary Lung Cancer; PTR-MS = Proton Transfer Reaction Mass Spectrometry; Ref. = Reference; SAW = surface acoustic wave; SIFT-MS = Selected Ion Flow Tube Mass Spectrometry; TB = pulmonary Tuberculosis; VAP = Ventilator Associated-Pneumonia; VOCs = Volatile Organic Compounds.

### Data synthesis and analysis

Evidence data were pooled by study design; studies using: 1) single VOCs in diagnosing pulmonary diseases (Table 2); 2) VOCs profiles in diagnosing pulmonary diseases (Table 3); and 3) VOCs profiles in differential diagnosing pulmonary diseases (Table 4).

**Table 2:** Studies using single VOCs for the diagnosis of various pulmonary diseases (diseased vs. healthy controls)

**Author (year)**	**Marker**	**Disease**	**N**	**Value**	**Unit**	**Diff.**	**Value marker**	**Unit**	**Controls**	**N**	**p-value**	**Ref.**
Lärstad (2007)	Ethane	Asthma	13	N.S.		=	N.S.		Controls	14	p > 0.05	[21]
	Pentane			N.S.		=	N.S.				p > 0.05	
	Isoprene			113	ppb^∞^	<	143	ppb^∞^			p < 0.05	
Olopade (1997)	Pentane	Acute asthma	12	8.4 ± 2.9	nmol/L*	>	2.6 ± 0.2	nmol/L*	Controls	17	p < 0.05	[23]
	Pentane	Stable asthma	11	3.6 ± 0.4	nmol/L*	=	2.6 ± 0.2	nmol/L*	Controls	17	p > 0.05	
Paredi (2000)	Ethane**	Steroid naïve asthma	12	2.06 ± 0.30	ppb*	>	0.88 ± 0.09	ppb*	Controls	14	p < 0.01	[24]
Paredi (2000)	Ethane**	Steroid naïve COPD	12	2.77 ± 0.25	ppb*	>	0.88 ± 0.09	ppb*	Controls	14	p < 0.05	[30]
Barker (2006)	Pentane**	CF	20	0.36 (0.24-0.48)	ppb^#^	>	0.21 (0.13-0.29)	ppb^#^	Controls	20	p < 0.05	[34]
	Dimethyl Sulphide**			3.89 (2.24-5.54)	ppb^#^	<	7.58 (5.73-9.43)	ppb^#^			p < 0.01	
	Ethane**			0.39 (−0.04-0.82)	ppb^#^	=	0.10 (−0.25-0.44)	ppb^#^			p > 0.05	
	Propane, methanol, ethanol, acetone, isoprene, benzene, toluene, limonene			-		=	-				p > 0.05	
Kamboures (2005)	Carbonyl sulphide**	CF	20	- 110 ± 60	pptv^#^	>	- 250 ± 20	pptv^#^	Controls	23	p < 0.001	[37]
	Dimethyl sulphide			4,780 ± 1,350	pptv^#^	=	3,920 ± 680	pptv^#^			p > 0.05	
	Carbon sulphide**			26 ± 38	pptv^#^	>	- 17 ± 15	pptv^#^			p < 0.05	
McGrath (2000)	Isoprene	CF during exacerbation	12	125 ± 23	pmol·min·kg^-1^*	<	164 ± 20	pmol·min·kg^-1^*	Controls	12	p < 0.05	[38]
	Isoprene	CF after exacerbation	12	188 ± 23	pmol·min·kg^-1^*	=	164 ± 20	pmol·min·kg^-1^*	Controls	12	p > 0.05	
Paredi (2000)	Ethane**	Steroid naïve CF	23	1.99 ± 0.20	ppb*	>	0.82 ± 0.09	ppb*	Controls	14	p < 0.05	[39]
Shestivska (2011)	Methyl thiocyanate	CF	28	7 (2–21)	ppbv^~^	=	8 (5–8)	ppbv°°	Controls	9	p > 0.05	[40]
Bajtarevic (2009)	Isoprene	LC	220	81.5	ppb^∞^	<	105.2	ppb^∞^	Controls	441	p < 0.01	[41]
	Acetone			458.7	ppb^∞^	<	627.5	ppb^∞^			p < 0.01	
	Methanol			118.5	ppb^∞^	<	142.0	ppb^∞^			p < 0.05	
Buszewski (2012)	Acetone	LC	29	34.57-390.60	ppb°	?	44.20-531.45	ppb°	Controls	44	p < 0.05	[42]
	Benzene			1.29-3.82	ppb°	?	1.38-14.97	ppb°			p < 0.05	
	Butanal			1.32-2.55	ppb°	>	1.35-1.87	ppb°			p < 0.01	
	2-Butanone			1.35-2.86	ppb°	?	1.35-3.18	ppb°			p < 0.01	
	Ethyl acetate			3.98-22.89	ppb°	>	1.12-8.22	ppb°			p < 0.01	
	Ethyl benzene			1.45-3.16	ppb°	?	2.22-18.38	ppb°			p < 0.01	
	2-Pentanone			3.25-8.77	ppb°	>	1.80-4.11	ppb°			p < 0.01	
	Propanal			1.56-3.74	ppb°	>	1.56-3.44	ppb°			p < 0.01	
	1-Propanol			4.37-13.15	ppb°	>	N.S.	ppb°			p < 0.01	
	2-Propanol			3.32-7.19	ppb°	>	3.21-4.17	ppb°			p < 0.01	
	2-Propenal			6.84-94.36	ppb°	>	5.10-9.57	ppb°			p < 0.05	
	Other VOCs			N.S.	ppb°	=	N.S.	ppb°			p > 0.05	
Crohns (2009)	Pentane**	LC	11	1.73 (1.05-2.86)	ng/L^#^	>	0.83 (0.61-1.13)	ng/L^#^	Controls	30	p < 0.05	[43]
Fuchs (2010)	Pentanal**	LC	12	0.019 (0.011-0.031)	nmol/Lˆ	>	0.002 (0.000-0.011)	nmol/Lˆ	Controls	12	p < 0.05	[45]
	Hexanal**			0.010 (0.008-0.026)	nmol/Lˆ	>	0.000 (0.000-0.001)	nmol/Lˆ			p < 0.05	
	Octanal**			0.052 (0.026-0.087)	nmol/Lˆ	>	0.011 (0.004-0.028)	nmol/Lˆ			p < 0.05	
	Nonanal**			0.239 (0.128-0.496)	nmol/Lˆ	>	0.033 (0.021-0.096)	nmol/Lˆ			p < 0.05	
	Acetaldehyde**, Propanal, butanal**, heptanal, decanal**			-		=	-				p > 0.05	
Kischkel (2010)	Dimethyl sulphide**	LC	31	0.27 (0.00-0.27)	nmol/Lˆ	<	0.30 (0.00-0.31)	nmol/Lˆ	Controls	31	p < 0.01	[48]
	Dimethyl formamide**			1855 (0.00-3340.88)	(counts)ˆ	>	0.00 (0.00-2954.13)	(counts)ˆ			p < 0.05	
	Butane**			0.00 (0.00-0.11)	nmol/Lˆ	>	0.18 (0.00-0.52)	nmol/Lˆ			p < 0.01	
	Butanal**			1.07 (0.38-3.51)	nmol/Lˆ	>	0.32 (0.00-1.40)	nmol/Lˆ			p < 0.001	
	Other VOCs (N = 38)			N.S.			N.S				p > 0.05	
Poli (2005)	2-Methylpentane	NSCLC	36	139.5 (65.7-298.8)	10^-12^Mˆ	>	27.7 (3.4-50.3)	10^-12^Mˆ	Controls	50	p < 0.001		[57]
	Pentane			647.5 (361.3-1112.5)	10^-12^Mˆ	>	268.0 (107.7-462.7)	10^-12^Mˆ			p < 0.001		
	Ethylbenzene			24.0 (13.6-32.6)	10^-12^Mˆ	>	13.6 (10.8-15.1)	10^-12^Mˆ			p < 0.01		
	Xylenes			68.9 (43.6-108.4)	10^-12^Mˆ	>	31.1 (21.1-56.4)	10^-12^Mˆ			p < 0.001		
	Trimethylbenzene			14.9 (9.3-22.1)	10^-12^Mˆ	>	6.2 (4.7-11.0)	10^-12^Mˆ			p < 0.01		
	Toluene			158.8 (118.7-237.5)	10^-12^Mˆ	>	80.8 (58.9-140.0)	10^-12^Mˆ			p < 0.001		
	Benzene			94.5 (62.2-132.2)	10^-12^Mˆ	>	44.7 (27.7-68.6)	10^-12^Mˆ			p < 0.001		
	Decane			568.0 (277.9-1321.6)	10^-12^Mˆ	>	208.7 (14.3-405.5)	10^-12^Mˆ			p < 0.001		
	Octane			61.0 (22.4-112.9)	10^-12^Mˆ	>	20.2 (4.0-50.8)	10^-12^Mˆ			p < 0.001		
	Pentamethylheptane			2.5 (1.2-9.7)	10^-12^Mˆ	>	0.9 (0.1-2.6)	10^-12^Mˆ			p < 0.001		
	Isoprene, heptane, styrene			-		=	-				p > 0.05		
Poli (2008)	2-Methylpentane	NSCLC (3 yrs after surgery)	10	87.9 (35.5-278.9)	10^-12^Mˆ	>	27.7 (3.4-50.3)	10^-12^Mˆ	Controls	50	p < 0.05	[58]	
	Pentane			1569.0 (497.9-3214)	10^-12^Mˆ	>	268.0 (107.7-462.7)	10^-12^Mˆ			p < 0.001		
	Ethylbenzene			46.4 (38.6-90.9)	10^-12^Mˆ	>	13.6 (10.8-15.1)	10^-12^Mˆ			p < 0.001		
	Xylenes			56.2 (38.9-80.4)	10^-12^Mˆ	>	31.1 (21.1-56.4)	10^-12^Mˆ			p < 0.05		
	Trimethylbenzene			15.3 (11.7-22.3)	10^-12^Mˆ	>	6.2 (4.7-11.0)	10^-12^Mˆ			p < 0.001		
	Toluene			297 (202.6-297.0)	10^-12^Mˆ	>	80.8 (58.9-140.0)	10^-12^Mˆ			p < 0.001		
	Pentamethylheptane			8.8 (2.2-15.2)	10^-12^Mˆ	>	0.9 (0.1-2.6)	10^-12^Mˆ			p < 0.001		
	Isoprene			678.9 (359.8-1111.0)	10^-12^Mˆ	<	3789 (1399–6589)	10^-12^Mˆ			p < 0.01		
	Benzene, Heptane, Octane, Styrene			-		=	-				p > 0.05		
Preti (1988)	O-toluidine	LC	10	N.S		>	N.S		Controls	16	p < 0.05	[60]	
	Aniline			N.S		=	N.S				p > 0.05		
Rudnicka (2011)	Propane	LC	23	3.19-9.74	ppb°	>	3.45-5.96	ppb°	Controls	30	p < 0.05	[61]	
	2-Propenal			N.S		?	N.S				p < 0.05		
	Carbon disulfide			N.S		?	N.S				p < 0.05		
	Isopropyl alcohol			N.S		?	N.S				p < 0.05		
	Ethylbenzene			1.45–3.16	ppb°	<	2.22–18.38	ppb°			p < 0.05		
	Styrene			N.S		?	N.S				p < 0.05		
	Other VOCs (N = 49)			N.S		=	N.S				p > 0.05		
Skeldon (2006)	Ethane**	LC	12	0.7 (0–7.6)	ppb^~^	=	1.9 (0–10.54)	ppb^~^	Controls	12	p > 0.05	[62]	
Song (2010)	1-Butanol**	NSCLC	43	6.36 (12.93)	ng/Lˆ	>	2.18 (2.06)	ng/Lˆ	Controls	41	p < 0.001	[63]	
	3-Hydroxy-2-butanone**			8.28 (11.52)	ng/Lˆ	>	1.29 (2.01)	ng/Lˆ			p < 0.001		
Ulanowska(2011)	Ethanol**	LC	137	466.9 (12.8-1520.1)	ppb°°	>	188.5 (4.5-479.5)	ppb°°	Controls	86	p < 0.05	[65]	
	Acetone**			358.6 (112.3-2653.7)	ppb°°	>	225.7 (41.6-753.4)	ppb°°			p < 0.05		
	Butane**			90.3 (6.1-421.3)	ppb°°	>	56.2 (5.2-165.7)	ppb°°			p < 0.05		
	Dimethyl sulphide**			11.9 (6.3-18.5)	ppb°°	>	9.3 (5.3-19.3)	ppb°°			p < 0.05		
	Isoprene**			100.3 (19.2-295.5)	ppb°°	>	70.8 (19.5-200.5)	ppb°°			p < 0.05		
	Propanal**			7.8 (5.5-33.8)	ppb°°	>	6.9 (5.6-9.1)	ppb°°			p < 0.05		
	1-Propanol**			54.8 (5.4-473.3)	ppb°°	>	6.6 (N.S.)	ppb°°			p < 0.05		
	2-Pentanone**			7.5 (4.4-53.2)	ppb°°	>	4.8 (4.6-5.1)	ppb°°			p < 0.05		
	Furan**			4.7 (3.1-7.0)	ppb°°	>	3.7 (3.0-5.3)	ppb°°			p < 0.05		
	o-Xylene**			22.1 (7.6-95.2)	ppb°°	>	17.4 (6.2-30.8)	ppb°°			p < 0.05		
	Ethylbenzene**			19.6 (4.6-89.3)	ppb°°	>	10.4 (8.6-14.0)	ppb°°			p < 0.05		
	Other VOCs (N ≈ 20)			-		=	-				p > 0.05		
Wehinger (2007)	Formaldehyde	PLC	17	7.0 (15.5)	ppbˆ	>	3.0 (1.9)	ppbˆ	Controls	170	p < 0.001	[66]	
	Propanol			244.1 (236.2)	ppbˆ	>	94.1 (55.2)	ppbˆ			p < 0.001		
	Isoprene			52.1 (26.7)	ppbˆ	<	81.8 (56.1)	ppbˆ			p < 0.01		
	Acetone, o-Toluidine			-		=	-				p > 0.05		
Gennaro (2010)	Cyclohexane**	MPM	13	251.79 (84%)	ng/L^∂^	>	33.08 (58%)	ng/L^∂^	Controls	13	p < 0.05	[70]	
	Other VOCs (N = 19)			-		=	-				p > 0.05		
Syhre (2009)	Methyl nicotinate	TB	10	N.S		>	N.S		Controls	10	p < 0.01	[80]	
Chambers (2009)	2-Pentylfuran***	A. fumigatus	17	Sens: 77, Spec: 78	%	>	Not detected		Controls	14	N.S.	[71]	
Kanoh (2005)	Ethane**	ILD	34	8.5 ± 8.0	pmol/dL*	>	2.9 ± 1.0	pmol/dL*	Controls	16	p < 0.001	[75]	
Scholpp (2002)	Acetone	ARDS	13	50.0 (19.6-72.3)	nmol/Lˆ	=	33.2 (20.8-38.6)	nmol/Lˆ	Controls	10	p > 0.05	[81]	
	Isoprene			2.18 (1.1-3.89)	nmol/L^#^	<	5.99 (3.53-8.45)	nmol/L^#^			p < 0.05		
	n-Pentane			1.00 (0.26-1.72)	nmol/Lˆ	>	0.12 (0.10-0.16)	nmol/Lˆ			p < 0.05		
	n-Pentane	At risk ARDS	33	0.49 (0.30-0.99)	nmol/Lˆ	>	0.12 (0.10-0.16)	nmol/Lˆ	Controls	10	p < 0.05		
Schubert (1998)	Acetone	ARDS	19	149 (113–485)	nmol/m^2 ≈^	=	119 (52–270)	nmol/m^2≈^	No ARDS	18	p > 0.05	[82]	
		Isoprene			9.8 (8.2-21.6)	nmol/m^2 ≈^	<	21.8 (13.9-41.4)	nmol/m^2≈^			p < 0.05	
		n-Pentane			4.2 (3.7-9.3)	nmol/m^2 ≈^	=	5.1 (1.4-18.6)	nmol/m^2≈^			p > 0.05	

Data are presented as; *mean ± SEM or SD; ^#^mean (95% confidence interval);^∞^median; ˆmedian (25^th^-75^th^ percentile); ^~^median (range); ^≈^median (95% confidence interval); ° range; °° mean (range); ^∂^ median (relative standard deviation). ** Exhaled concentrations corrected for ambient concentrations (e.g. subtraction, VOCs filter). *** Sensitivity and specificity 2-Pentylfuran compared with gold standard (sputum). Diff. = Difference between diseased and controls: > elevated in diseased vs. controls, = no difference in diseased vs. controls, < decreased in diseased vs. controls. Abbreviations: A. fumigatus = Aspergillus fumigatus; ARDS = Acute Respiratory Distress Syndrome; CF = Cystic Fibrosis; COPD = Chronic Obstructive Pulmonary Disease; ILD = Interstitial Lung Disease (e.g. sarcoidosis, idiopathic pulmonary fibrosis, cryptogenic organizing pneumonia); LC = Lung Cancer; MPM = Malignant Pleural Mesothelioma; N = Sample size; N.S. = Not Stated; NSCLC = Non-Small Cell Lung Cancer; PLC = Primary Lung Cancer; Ref. = Reference.

**Table 3:** Studies using VOCs profiles for the diagnosis of various pulmonary diseases (diseased vs. healthy controls)

**Author (year)**	**Disease**	**N**	**Discriminative**	**Controls**	**N**	**No. of markers**	Sensitivity/Specificity (%)*	**Ref.**
Caldeira (2011)	Asthma	35	+	Controls	15	28	CVV: 88%	[17]
Caldeira (2012)	Asthma	32	+	Controls	27	9	98/93	[18]
Dallinga (2010)	Asthma	63	+	Controls	57	8 to 22	89 - 100/95 - 100	[19]
Dragonieri (2007)	Mild asthma	10	+	Controls	10	N.S.	CVV: 100% (M-distance 5.32)	[10]
	Severe asthma	10	+	Controls	10	N.S.	CVV: 90% (M-distance 2.77)	
Fens (2009)	Asthma	20	+	Non-smoking controls	20	N.S.	CVV: 95% (p < 0.001)	[15]
	Asthma	20	+	Smoking controls	20	N.S.	CVV: 93% (p < 0.001)	
Ibrahim (2011)	Asthma	35	+	Non-smoking controls	23	15	CVV: 83% (PPV: 0.85, NPV: 0.89)	[20]
Montuschi (2010)	Asthma	27	+	Controls	24	N.S.	DP: 88%	[22]
Timms (2012)	Asthma	20	+	Controls	7	N.S.	CVV: 70% (p = 0.047)	[32]
	COPD	17	+	Controls	7	N.S.	M-distance: 3.601 (p < 0.01)	
Cristescu (2011)	Emphysema	43	-	(Former) smoking controls	161	1	AUC: 0.56 (CI: 0.45-0.66)	[26]
Basanta (2010)	COPD	20	+	Smoking controls	6	N.S.	88/81	[25]
Fens (2009)	COPD	30	+/−	Smoking controls	20	N.S.	CVV: 66% (p < 0.01)	[15]
	COPD	30	-	Non-smoking controls	20	N.S.	CVV: N.S.	
Hattesohl (2011)	COPD	23	+/−	Controls	10	N.S.	CVV: 68% (p < 0.001)	[28]
Hauschild (2012)	COPD	84	+	Controls	35	120	87 - 98/71 - 86	[29]
Phillips (2012)	COPD	119	+	Controls	63	N.S.	79/64	[31]
Van Berkel (2010)	COPD	50	+	Controls	29	6 to 13	98 - 100/88 - 100	[33]
	COPD (validation)	16	+	Controls (validation)	16	6	100/81	
Robroeks (2010)	CF	48	+	Controls	57	22	100/100	[14]
Bajtarevic (2009)	LC	65	+	Controls	31	15 to 21	71 - 80/100 - 100	[41]
D’Amico (2010)	LC	28	+	Controls	36	N.S.	85/100	[44]
Di Natale (2003)	LC	35	+	Controls	18	N.S.	100/94	[11]
Dragonieri (2009)	NSCLC	10	+	Controls	10	N.S.	CVV: 90% (M-distance 2.96)	[12]
Gaspar (2009)	LC	18	+	Controls	10	10	100/100	[46]
Gordon (1985)	LC	12	+	Controls	9	22	DP > 80%	[47]
Ligor (2009)	LC	65	+/−	Controls	31	8	51/100	[49]
Machado (2005)	LC	14	+	Controls	20	N.S.	CVV: 72% (M-distance: 3.25)	[13]
Mazzone (2007)	NSCLC	49	-	Controls	21	N.S.	57/78	[50]
Peng (2009)	LC	40	+	Controls	56	42	2 PCA clusters: 100% discrimination	[52]
Peng (2010)	PLC	30	+	Controls	22	33	2 PCA clusters: 100% discrimination	[53]
Phillips (2003)	PLC	67	+	Controls	41	9	85/81	[7]
Phillips (2007–2008)	PLC	193	+	Controls	211	16 to 30	85 - 85/80 - 81	[55], [56]
Poli (2010)	NSCLC	40	+	Controls	38	7	90/92	[59]
Steeghs (2007)	LC	11	+	Controls	57	2	AUC: 0.81	[64]
Westhoff (2009)	LC	32	+	Controls	54	23	100/100	[67]
Chapman (2012)	MPM	10	+	Controls	32	N.S.	90/91	[68]
Dragonieri (2012)	MPM	13	+	Controls	13	N.S.	CVV: 85% (p < 0.001)	[69]
Phillips (2007)	Patients suspected of TB	42	+	Controls	59	N.S. (≈7)	100/100	[77]
Phillips (2012)	Patients with TB	130	+/−	Controls	121	8	71/72	[79]

*Sensitivity/Specificity (in %), unless stated otherwise. AUC = Area Under the ROC Curve; CF = Cystic Fibrosis; CI = 95% Confidence interval; COPD = Chronic Obstructive Pulmonary Disease; CVV = Cross-Validated accuracy-Value; DP = Diagnostic Performance; LC = Lung Cancer; M-distance = Mahalanobis-distance; MPM = Malignant Pleural Mesothelioma; N = Sample size; NPV = Negative Predictive Value; N.S. = Not Stated; NSCLC = Non-Small Cell Lung Cancer; PCA = Principal Component Analysis; PLC = Primary Lung Cancer; PPV = Positive Predictive Value; Ref. = Reference; TB = pulmonary Tuberculosis.

**Table 4:** Studies using VOCs profiles for the differential diagnosis of various pulmonary diseases

**Author (year)**	**Disease I**	**N**	**Discriminative**	**Disease II**	**N**	**No. of markers**	Sensitivity/Specificity (%)*	**Ref.**
D’Amico (2010)	LC	28	+	Other lung diseases	28	N.S.	93/79	[44]
Dragonieri (2007)	Mild asthma	10	+/−	Severe asthma	10	N.S.	CVV: 65% (M-distance 1.23)	[10]
Fens (2009)	Asthma	20	+	COPD	30	N.S.	CVV: 96% (p < 0.001)	[15]
Fens (2011) **	Fixed Asthma	21	+	COPD	40	N.S.	85/90 (CVV: 88%, p < 0.001)	[27]
	Classic Asthma	39	+				91/90 (CVV: 83%, p < 0.001)	
Ibrahim (2011)	Controlled Asthma	17	+	Uncontrolled asthma	18	13	89/88 (PPV: 0.89, NPV: 0.88)	[20]
Timms (2012)	Asthma	20	+	COPD	17	N.S.	CVV: 70% (p < 0.05)	[32]
	Asthma	11	+	Asthma with GER	9		CVV: 85% (p < 0.05)	
	COPD	8	+/-	COPD with GER	9		CVV: 65 (p < 0.05)	
Hattesohl (2011)	COPD without AAT deficiency	23	+/-	COPD with AAT deficiency	10	N.S.	CVV: 58% (M-distance: 2.27)	[28]
Dragonieri (2009)	LC	10	+	COPD	10	N.S.	CVV: 85% (M-distance: 3.73)	[12]
Machado (2005)	LC (validation)	14	+/-	No LC	62	N.S.	71/92	[13]
Mazzone (2007)	LC	49	+/−	No LC	94	N.S.	73/72	[50]
Mazzone (2012)	NSCLC	83	+	No LC	137	N.S.	70/86	[51]
	Adenocarcinoma	50	+	No LC	137		80/86	
	Squamous cell	23	+	No LC	137		91/73	
	Adenocarcinoma	50	+	Squamous cell	22		90/83	
Phillips (1999)	LC	60	+/−	No LC	48	22	72/67	[54]
Phillips (2003)	MLC	15	-	No MLC	91	9	67/37	[7]
Poli (2005)	NSCLC	36	+	No LC	110	13	72/94	[57]
Chapman (2012)	MPM	10	+	ARD	18	N.S.	90/83	[68]
Dragonieri (2012)	MPM	13	+	No MPM	13	N.S.	CVV: 81% (p < 0.001)	[69]
Hanson (2005)	+ VAP score	19	+	- VAP score	19	N.S.	R^2^ (to standard): 0.81 (p < 0.0001)	[72]
Hockstein (2004)	VAP	13	+	No VAP	12	N.S.	CVV: >80%	[73]
Hockstein (2005)	+ VAP score	15	+/−	- VAP score	29	N.S.	CVV: 66-70%	[74]
Kolk (2012)	TB	50	+	No TB	50	7	72/86	[76]
	TB (validation)	21	+	No TB	50	7	62/84	
Phillips (2007)	TB	23	+	No TB	19	N.S. (≈14)	96/79	[77]
Phillips (2010)	TB	N.S.	+	No TB	N.S.	N.S. (≈10)	84/65	[78]

*Sensitivity/Specificity (in %), unless stated otherwise. AAT deficiency = Alpha 1-antitrypsin deficiency; ARD = benign asbestos-related diseases; Classic asthma = Asthmatics with reversible airway obstruction; COPD = Chronic Obstructive Pulmonary Disease; CVV = Cross-Validated accuracy-Value; DP = Diagnostic Performance; Fixed asthma = Asthmatics with fixed airway obstruction; GER = Gastro-Esophageal Reflux; LC = Lung Cancer; M-distance = Mahalanobis-distance; MLC = Metastatic Lung Cancer; MPM = Malignant Pleural Mesothelioma; N = Sample size; NPV = Negative Predictive Value; N.S. = Not Stated; NSCLC = Non-Small Cell Lung Cancer; PPV = Positive Predictive Value; R^2^ = Coefficient of determination; TB = pulmonary Tuberculosis; VAP = Ventilator Associated-Pneumonia. ** External validation study of Fens 2009.

## Results

### Description of included studies

Seventy-three studies were included of which the characteristics are provided in Table 1. In total, nine studies described VOCs in asthma, seven in COPD, seven in CF, four compared asthma with COPD or CF, thirty-four in thoracic cancer (of which 6 studies included COPD patients in the control group), and twelve studies described VOCs in other pulmonary diseases. A total of 2,973 healthy controls and 3,952 patients were investigated; 417 asthmatic patients, 527 COPD patients, 188 CF patients, 1,575 lung cancer patients, 33 malignant pleural mesothelioma (MPM) patients, 139 subjects with an abnormal chest radiograph, 579 subjects suspected for pulmonary tuberculosis, and 494 patients with other (pulmonary) diseases (e.g. sarcoidosis, acute respiratory distress syndrome). Various techniques were described to collect and analyze exhaled VOCs. The most commonly used technique was gas chromatography (N = 50), usually combined with MS or FID. Fifteen studies analyzed VOCs using an eNose, whilst thirteen studies used PTR-MS, SIFT-MS, IMS, laser spectroscopy, colorimetric sensor array, and/or GNPs (some studies used multiple techniques). Forty-five studies were conducted in the last five years. An overview of findings per study can be found in Tables 2, 3, 4. The most important findings are summarized below.

### Volatile organic compounds in asthma

Several studies found that an accurate asthma diagnosis was possible using profiles of VOCs (Table 3). Dragonieri and Fens et al. demonstrated that a VOCs profile could correctly classify asthmatic patients when using an eNose [10,15]. Moreover, Montuschi and colleagues demonstrated that VOCs profiling using an eNose had higher diagnostic performance for asthma than exhaled nitric oxide or lung function [22]. Dallinga and Caldeira et al. demonstrated that VOCs profiling was able to accurately distinguish children with asthma from controls [17-19]. With respect to differential diagnosis, it was demonstrated that an eNose VOCs profile was able to discriminate between asthma and COPD patients (Table 4) [15,32]. An external validation study demonstrated that not only ‘classical’ asthmatic patients (with reversible airway obstruction) but also asthmatic patients with fixed airway obstruction could be distinguished from COPD patients [27]. As these latter two groups usually have similar symptoms and overlapping spirometry, differential diagnosis is often difficult. These findings imply that VOCs profiling is of additional value in the differential diagnosis of asthma and COPD.

Next to diagnosing asthma, VOCs might be useful in the assessment of asthma severity and control. Paredi et al. found elevated levels of exhaled ethane in steroid-naïve asthmatics compared to steroid-treated asthmatics (Table 2). Furthermore, ethane was higher in patients with severe asthma (FEV_1_ < 60%), compared to patients with mild asthma (FEV_1_ > 60%) [24]. In contrast, Dragonieri et al. reported that it was not possible to adequately distinguish mild and severe asthmatics using an eNose profile [10]. Regarding asthma control, higher exhaled pentane levels were found in asthmatic patients with an exacerbation compared to controls. Once the asthma exacerbation subsidized, pentane levels decreased to levels comparable to controls [23]. Moreover, Ibrahim demonstrated that VOCs profiles were able to diagnose sputum eosinophilia and identify patients with poor disease control [20]. Taken together, VOCs profiling might be useful for an asthma diagnosis, for differentiating asthma from COPD, and for assessing asthma control. The usefulness of VOCs profiles in assessing disease severity still needs to be established.

### Volatile organic compounds in chronic obstructive pulmonary disease

Many COPD patients are diagnosed at an advanced stage of the disease, when benefits of interventions such as smoking cessation and drug therapy are less pronounced. An early diagnosis of COPD would be an advantage. Multiple research groups demonstrated that VOC profiles could accurately differentiate COPD patients from healthy (non-) smokers [25,29,31,33]. In contrast, others found a limited performance of VOCs profiles to differentiate COPD patients from (former) smokers [15,26]. Hattesohl et al. demonstrated that eNose derived VOCs profiles were not different between COPD patients with and without an alpha 1-antitrypsin (AAT) deficiency, after internal cross-validation. Moreover, cross-validated VOCs profiles of AAT deficiency patients did not differ after human recombinant AAT therapy [28].

Next to diagnostic purposes, VOCs might be useful to monitor severity and inflammation status in COPD patients. Elevated levels of ethane were found in steroid-naïve COPD patients and patients with low FEV_1_ values compared to steroid-treated patients and patients with higher FEV_1_ values [30]. Fens et al. demonstrated that VOCs profiles were associated with both cell counts and sputum markers of inflammatory cell activation (eosinophilic vs. neutrophilic) in COPD patients [83]. These findings indicate that VOCs profiles might monitor both type and activity of airway inflammation.

### Volatile organic compounds in cystic fibrosis

In CF, there is less need for a new diagnostic tool as the sweat chloride test and genetic screening serve as gold standards. However, there is need for new tools regarding early detection of *Pseudomonas (P.) aeruginosa* and prediction and follow-up of exacerbations. Robroeks et al. demonstrated that a VOCs profile could accurately discriminate between CF patients with and without *P. aeruginosa* colonization [14]. Gilchrist et al. showed that exhaled hydrogen cyanide (a marker of *P. aeruginosa*) was elevated in CF children with *P. aeruginosa* colonization compared to CF children without colonization [36]. Accordingly, Enderby et al. demonstrated that exhaled hydrogen cyanide was elevated in children with CF compared to children with asthma [35]. Kamboures et al. demonstrated elevated levels of exhaled sulphides (produced by bacteria such as *P. aeruginosa)* in CF patients compared to controls [37]. In contrast, Shestivska et al. could not demonstrate different levels of exhaled methyl thiocyanate (also a marker of *P. aeruginosa*) in CF patients and controls [40].

Regarding monitoring disease control, McGrath et al. demonstrated that CF patients with an acute exacerbation had lower levels of exhaled isoprene compared to controls [38]. When these patients were treated with antibiotics, their isoprene levels increased to normal [38]. Moreover, elevated ethane levels were found in steroid-naïve CF patients compared to steroid-treated patients [39]. In addition, elevated pentane levels were found in CF patients with an exacerbation [34]. These data demonstrate that VOCs profiling can be useful for assessment and follow-up of exacerbations, and for a rapid detection of *P. aeruginosa* in CF patients.

### Volatile organic compounds in thoracic oncology

The majority of lung cancer (LC) patients studied had non-small cell lung cancer (Table 1). Several studies demonstrated that a combination of VOCs, identified by GC-MS, could differentiate LC patients from controls [7,41,46,47,49,52,53,55,56,59]. In general, the number of VOCs per model ranged from 7 to 33, with a sensitivity of 50-100% and a specificity of 80-100% (Table 3). These studies, together with studies investigating single VOCs, revealed that the discriminative VOCs were predominantly alkanes (e.g. pentane, butane, propane), alkane derivates (e.g. propanol, multiple aldehydes) and benzene derivates (e.g. ethyl-, propylbenzene) (Table 2) [42,43,45,48,57,59-63,65,66]. Although most VOCs levels were elevated, certain levels (e.g. of isoprene) were decreased in patients compared to controls [41,58,66]. The diagnostic potential of VOCs profiles in LC was also demonstrated by groups that used eNose and other sophisticated techniques [11-13,44,51-53,64,67]. Moreover, breath profiles were different in patients with dissimilar histology (adenocarcinoma vs. squamous cell carcinoma) [51]. Besides, Peng et al. demonstrated distinct VOCs profiles in patients with lung, colon, breast, and prostate cancer [53]. The important findings of VOC signatures of different cancer types, need to be confirmed in wider clinical studies.

Multiple studies investigated the potential of VOCs to discriminate between LC and other pulmonary diseases. Not single compounds (such as ethane), but a combination of multiple VOCs was able to distinct LC patients from patients with non-cancer pulmonary diseases (such as COPD, pleurisy, idiopathic fibrosis) with a reasonable accuracy (Table 4) [12,13,44,50,57,62]. Phillips et al. demonstrated that primary LC could be reasonably diagnosed in subjects with an abnormal chest radiograph [7,54]. However, VOCs had limited predictive value to stage LC patients [7,63,84].

Two studies described the potential of VOCs in evaluating treatment in LC patients. Poli et al. demonstrated that VOCs levels, except for isoprene, were unaffected one month after surgical resection of the tumor [58]. After three years, several VOCs either increased (e.g. pentane) or decreased (e.g. isoprene) compared to baseline [58]. However, most post-surgical VOCs levels remained higher compared to levels of controls. Likewise, Crohns et al. were not able to detect changes of pentane levels after radiotherapy, although they did demonstrate that higher pre-treatment levels predicted better survival [43].

Malignant pleural mesothelioma (MPM) is a rare tumor mainly caused by asbestos exposure. VOCs profiles were able to diagnose MPM in a group of subjects with long-term professional asbestos exposure [68,69]. Moreover, de Gennaro et al. distilled cyclohexane as possible marker of MPM [70].

Smoking status can be an important influencing factor, especially in patients with LC and COPD. Smoke contains profuse amounts of VOCs and is associated with alterations in exhaled VOCs patterns. As high background of external VOCs caused by smoking can influence the accuracy of the diagnostic profile, most studies performed in LC and COPD took smoking status into account in their analysis [15,25,27,31,33,43,50,53-55,59,65].

### Volatile organic compounds in other pulmonary diseases

VOCs were also studied in critically ill patients with acute respiratory distress syndrome (ARDS). Lower isoprene levels and elevated pentane levels were reported in ARDS patients compared to controls [81,82]. These findings are in line with the findings that critically ill patients with ventilator-associated pneumonia (VAP) had decreased isoprene levels and increased pentane levels compared to patients without pneumonia [82]. In addition, VOCs profiles generated with the eNose had potential to diagnose this form of pneumonia [72-74].

Kanoh et al. demonstrated that exhaled ethane was elevated in patients with an interstitial lung disease (including sarcoidosis and idiopathic pulmonary fibrosis) compared to controls, with highest levels in those with an active and progressive disease [75]. A small VOC, 2-pentylfuran, was commonly present in breath of patients with a chronic pulmonary disease (including asthma and CF) with *Aspergillus fumigatus* in their respiratory specimens, whereas this VOC was not detected in breath of controls [71]. Syhre et al. demonstrated elevated levels of exhaled methyl nicotinate (a volatile metabolite produced by *M. tuberculosis*) in patients with pulmonary tuberculosis (TB) compared to healthy controls [80]. In a group of patients with suspicion of TB, VOC patterns were able to distinguish patients with TB from those without active TB and healthy controls with a reasonable accuracy (Table 4) [76-79].

## Discussion

### Conclusions from this review

A substantial increase in clinical studies on VOCs in pulmonary diseases was observed in the last decade. Initial studies on VOCs identified biomarkers in a traditional way by focusing on single compounds based on biological insight. Levels of several VOCs were demonstrated to be distinct in people with a pulmonary disease compared to controls. These markers mainly included alkanes for asthma and COPD; alkanes, alkenes and alkene-derivates for CF and analogous compounds plus benzene derivates and aldehydes in lung cancer. Due to overlap in markers, one may argue that a disease specific biomarker is not discovered yet. For example, ethane was not only elevated in asthmatic patients, but also in COPD and CF patients. Similarly, decreased levels of isoprene were found in both children with CF, asthmatic patients, lung cancer patients and in patients with ARDS. Despite the lack of a single discriminative biomarker, these studies did demonstrate that exhaled breath of patients with a particular lung disease is distinct from healthy controls. This finding evolved in a new hypothesis that pulmonary diseases are characterized by a distinctive breath-print that is not based on single markers, but on a profile of numerous VOCs. Instead of a knowledge based strategy, recent studies mainly support an inductive strategy to discover disease-specific VOCs profiles. Owing to recent technical and analytical advancements, hundreds of VOCs can be analyzed to characterize the breath-print of a pulmonary disease. Various research groups demonstrated that, either by using the eNose or GC, VOCs profiles of patients with several pulmonary diseases could be well distinguished from VOCs profiles of controls. Moreover, distinct VOCs profiles were found in patients with dissimilar pulmonary diseases. These promising results pave the way for the development of a non-invasive diagnostic tool based on exhaled VOCs.

### Potential applications and advantages of using VOCs into clinical practice

Although current research mainly focused on the diagnostic potential of VOCs, there are multiple other conceivable applications of VOCs in the field of pulmonary diseases, such as:

(early) Diagnosing of pulmonary diseases (e.g. early asthma diagnosis in children).

Differential diagnosing (e.g. asthma versus COPD).

Phenotyping within a pulmonary disease (e.g. wheezing phenotypes in children).

Monitoring disease severity and control.

Predicting exacerbations and prognosis of a disease.

Evaluating treatment/surgery (e.g. checking compliance with prescribed medication).

Screening for different diseases in population based studies (e.g. predicting risk).

The advantages of VOCs profiling are evident. Although VOCs can have an exogenous origin, numerous VOCs are formed within the airways as a result of local inflammatory or neoplastic processes. Therefore, the analysis of exhaled VOCs can serve as a direct measure of lung status. Nevertheless, since VOCs are blood-borne they can also reflect other processes in the body and thus may assess different body functions in a flexible manner. Secondly, collection of breath samples is safe, non-invasive and easy to perform even in children and more severe patients. Breath collection does not require skilled medical staff and obtaining large quantities or repeated measurements are not as bothersome for patients compared to e.g. blood sampling, sputum induction or bronchoalveolar lavages. Moreover, the matrix of exhaled breath is less complex than blood or urine, eliminating the need for complicated work-up of samples. Finally, techniques to measure VOCs, such as GC-MS, are very sensitive to detect compounds and techniques such as the IMS allow real-time measurement of compounds in the body.

### Challenges before VOCs can be used into clinical practice

Although VOCs profiling is a potential clinical tool, considerable work needs to be done before it can be applied into clinical practice. An important step that needs to be taken is extensive validation of the current available VOCs profiles. Next to (external) validation, standardization in collecting and analyzing VOCs is necessary to enhance inter-laboratory comparability. Due to the heterogeneity of the included studies (in study design, sampling and analytical techniques) a variety of results is reported, making it difficult to draw firm conclusions or to calculate an algorithm for the most important compounds in the diverse pulmonary diseases. In the standardization procedure, the influence of potential confounders needs to be explored before considering VOCs as a clinical tool [48]. As described before, exhaled VOCs can arise from various endogenous and exogenous sources. Consequently, numerous environmental-, subject- and analytical factors can influence the exhaled VOCs pattern (Table 5). For example, air pollution, smoking, eating, drinking and medication use can considerably affect the composition of exhaled VOCs [85]. Also the presence of bacteria can alter exhaled VOCs patterns (hence breath analysis could be used to identify bacterial infections and bacterial-induced diseases). This suggests that these factors should be taken into account when constructing a diagnostic tool on basis of VOCs patterns. Moreover, potential confounders on analytical level should be carefully studied. For example, during the offline-procedure of exhaled VOCs collection, Tedlar bags could release VOCs into the collected breath, and storage onto Tenax columns can disturb the composition of the breath. As the pool of exhaled VOCs arises from multiple endogenous and exogenous sources, the analysis of background samples and standardization of the analysis per technique is necessary. A list of recommendations for measurements composed by experts, as was provided for markers in exhaled breath condensate, will facilitate standardization [86]. Thirdly, more insight is needed in the physiological meaning and biochemical origin of endogenous formed VOCs. However, this might be difficult since, as described before, the origin of VOCs is blood-borne and therefore can be the result of widely different biochemical pathways (so the previously mentioned advantage of VOCs is a disadvantage as well). Fourthly, more research is needed on the potential of VOCs in differential diagnosis and monitoring purposes. Besides these four major ‘missions’, others aspects of VOCs assessment and analysis can be improved. Although breath samples are easy to collect, the analysis of VOCs is still quite cumbersome and time-consuming and requires trained personnel. Moreover, further refinement of sampling techniques, exploring advanced statistical techniques on the multi-data of VOCs to build diagnostic and prognostic models, and developing new tools that combine the strengths of the eNose (cheap, time efficient), IMS (real-time), and GC-MS (sensitive, compound identification) will facilitate the introduction of VOCs into clinical practice.

**Table 5:** Factors that can influence exhaled VOCs in pulmonary diseases

**Source**	**Factor**
Environment	Ambient VOCs (e.g. by air pollution)
	Temperature of environment
	Humidity of inhaled and exhaled air
	Season
Subject	Clinical characteristics: e.g. age, gender, weight, length
	Nutrition
	Tobacco smoking
	Medication use
	Circadian rhythm
	Non-pulmonary chronic diseases (liver impairment, diabetes, presence of bacteria)
	Breathing pattern: e.g. exhaled flow, minute ventilation, breath hold
	Overall lifestyle and physical condition
Analysis	Time and way of storage
	Pre-concentration
	Breath collection: mixed air or alveolar air
	Collection method: e.g. tedlar bags, metal containers
	Analytical method: e.g. eNose, GC-MS

## Conclusions

As the current available tools are not always fulfilling, there is an increasing interest in non-invasive measurement of exhaled VOCs to improve the diagnosis and management of pulmonary diseases. Due to the complex pathophysiology of most pulmonary diseases, current research mainly focused on profiles of VOCs rather than on individual compounds. Promising findings were reported on VOCs profiles that were able to accurately diagnose and monitor various pulmonary diseases. However, multiple constraints including validation and standardization need to be resolved before VOCs can be applied into clinical practice. The rapid progress that is currently made in the field of VOCs will facilitate the imminent introduction of VOCs profiling as a non-invasive, additional tool to assist in diagnosing and monitoring of pulmonary diseases.

## Abbreviations

AAT deficiency: Alpha 1-antitrypsin deficiency; A. fumigatus: Aspergillus fumigatus; ARD: Benign Asbestos-Related Diseases; ARDS: Acute Respiratory Distress Syndrome; CBD: Chronic pulmonary Beryllium Disease; CF: Cystic Fibrosis; COPD: Chronic Obstructive Pulmonary Disease; CVV: Cross-Validated accuracy-Value; DMS: Differential Mobility Spectrometry; eNose: electronic Nose; FID: Flame Ionization Detector; GC: Gas Chromatography; GER: Gastro-Esophageal Reflux; GNPs: Gold Nano Particles sensors; ILD: Interstitial Lung Disease; IMS: Ion Mobility Spectrometry; LC: Lung Cancer; MC: Medical Centre; M-distance: Mahalanobis-distance; MLC: Metastatic Lung Cancer; MPM: Malignant Pleural Mesothelioma; MS: Mass Spectrometry; NPV: Negative Predictive Value; NSCLC: Non-Small Cell Lung Cancer; OFD: On-Fiber-Derivatization; PCA: Principal Component Analysis; PLC: Primary Lung Cancer; PPV: Positive Predictive Value; PTR-MS: Proton Transfer Reaction Mass Spectrometry; SAW: Surface Acoustic Wave; SIFT-MS: Selected Ion Flow Tube Mass Spectrometry; TB: pulmonary Tuberculosis; VAP: Ventilator Associated-Pneumonia; VOCs: Volatile Organic Compounds.

## Competing interests

The authors declare that they have no competing interests.

## Authors’ contributions

KvdK and LvdS included the studies, extracted the data and drafted the manuscript. QJ, CPvS, and ED helped to draft the manuscript. All authors read and approved the final manuscript.
